# Hsa_circ_0013561 promotes epithelial-mesenchymal transition and tumor progression by regulating ANXA2 via miR-23b-3p in ovarian cancer

**DOI:** 10.1038/s41417-023-00686-z

**Published:** 2023-12-15

**Authors:** Jia Lv, Yijun Zhang, Mengying Yang, Lianqiao Qiao, Huihui Wang, Huici Jiang, Mingxu Fu, Jinlong Qin, Shaohua Xu

**Affiliations:** 1 Department of Obstetrics and Gynecology, Shanghai Fourth People’s Hospital, School of Medicine, Tongji University, Shanghai, China; 2 Department of Gynecology, Shanghai First Maternity and Infant Hospital, School of Medicine, Tongji University, Shanghai, China; 3 Department of Obstetrics and Gynecology, Shanghai Tenth People’s Hospital, School of Medicine, Tongji University, Shanghai, China

**Keywords:** Ovarian cancer, Biomarkers

## Abstract

Our preliminary experiment discovered that hsa_circ_0013561 was aberrantly expressed in OC. However, the underlying mechanism is unclear. The expression of hsa_circ_0013561 in OC cells and tissues was detected by RT-qPCR and fluorescence in situ hybridization. The effects of hsa_circ_0013561 on the proliferation and metastasis of OC were explored by functional experiments such as cell counting kit-8, transwell, and tumor xenograft models. To mechanistically understand the regulatory role of hsa_circ_0013561, bioinformatics analysis, Western blot, luciferase reporter assay, and a series of rescue experiments were applied. We found that the hsa_circ_0013561 expression was elevated in OC cells and tissues, and was correlated with metastasis formation. Downregulation of hsa_circ_0013561 suppressed the proliferation and migration of OC cells both in vitro and in vivo. Regarding the interactions of hsa_circ_0013561, the luciferase reporter assay verified that miR-23b-3p and Annexin A2 (ANXA2) were its downstream targets. MiR-23b-3p inhibition or ANXA2 overexpression reversed OC cell proliferation, migration, and epithelial-mesenchymal transition (EMT) post-hsa_circ_0013561 silencing. Moreover, ANXA2 overexpression also reversed OC cell migration, proliferation, and EMT after miR-23b-3p upregulation. Our data suggest that hsa_circ_0013561 increases the expression of ANXA2 by regulating miR-23b-3p competitively, resulting in EMT and metastasis of OC. Thus, hsa_circ_0013561 may serve as a novel oncogenic biomarker for OC progression.

## Introduction

Ovarian cancer (OC) is a common malignancy worldwide and has the highest mortality rate among gynecological cancers [1]. Because OC is usually asymptomatic and insidious, about 3 out of 4 patients are diagnosed at an advanced stage [2]. Among these patients, tumor invasion and metastasis are inevitable, which is closely related to a poor prognosis. Although advances have been made in the management of OC over the past decades, the 5-year survival rate for advanced disease is only 29% [3]. As a result, it is of great clinical importance to identify the potential biomarkers and therapeutic targets in metastatic OC [4, 5].

Circular RNAs (circRNAs) are a special type of endogenous noncoding RNAs that have single‐stranded covalently closed loops [6, 7]. As lacking linear RNAs’ free ends such as 5’ caps or 3’ poly-A tails which are more vulnerable to RNase R [8], circRNAs are relatively stable in structure. CircRNAs are also conservative and widely distributed in organisms. In recent years, accumulating studies have reported on the biogenesis, expression, metabolism and function of circRNAs, especially on the regulatory role of circRNAs in cancer development and metastasis [7–9]. For instance, Li et al. previously confirmed that circ-ITGB6 induces cisplatin resistance of OC by forming a circRNA-protein-mRNA ternary complex [10]. Wang et al. showed that Circ-ATP2B4 functions as a competitive endogenous RNA (ceRNA) for miR-532-3p to regulate SREBF1, resulting in OC metastasis [11]. Although a large body of evidence indicates that circRNAs play a critical role in various cancers, the specific mechanism of circRNAs in OC progression remains largely unknown.

The goal of this study was to explore the functions of a novel circular RNA hsa_circ_0013561 in OC and investigate its underlying mechanisms in tumor progression. By conducting experiments in vitro and in vivo, we found that hsa_circ_0013561 is upregulated in OC cells and tissues, and is correlated with metastasis formation. Downregulation of hsa_circ_0013561 could suppress the proliferation and migration of OC cells. Mechanically, hsa_circ_0013561 increases the expression of Annexin A2 (ANXA2) by regulating miR-23b-3p competitively, resulting in epithelial-mesenchymal transition (EMT) and tumor progression. Therefore, hsa_circ_0013561 may serve as a promising oncogenic biomarker for OC progression.

## Methods

### Tissue chips and fluorescence in situ hybridization (FISH)

OC tissue chips (*n* = 96) were obtained from Zhuolibiotech (ZL-OVA961, Shanghai, China). Geneseed Biotech (San Diego, CA, USA) generated special probes to hsa_circ_0013561 (Dig-5′-AAGCCCAAGACCTAAAAGTGGTG-3′-Dig). For the FISH analysis, we detected signals using Cy3-conjugated anti-biotin antibodies (Jackson ImmunoResearch, West Grove, PA, USA) and counterstained nuclei using 4,6-diamidino-2-phenylindole (DAPI; Yeasen Biotechnology, Shanghai, China) for 15 min at room temperature. We took photographs using a Zeiss LSM 700 confocal microscope (Carl Zeiss GmbH, Oberkochen, Germany).

### Cell culture

We purchased four human OC cell lines (SKOV3, ES-2, OVCAR-3, and A2780) and one normal epithelial cell line (IOSE80) from the Chinese Academy of Sciences Cell Bank (Shanghai, China). We cultivated cells in Dulbecco’s modified eagle medium (DMEM, Gibco, Gaithersburg, MD, USA) with 10% fetal bovine serum (FBS, Gibco), 50 μg/mL streptomycin, and 50 μg/mL penicillin at 37 °C with 5% CO_2_.

### RNA overexpression or interference

MiR-23b-3p inhibitor (final small interfering RNA (siRNA) concentration: 20 nM), miR-23b-3p mimic (microRNA (miRNA) mimic: 100 nM, mature miRNA for miR-23b-3p: 5′-UAGCAGCACGUAAAUAUUGGCG-3′), hsa_circ_0013561 silencing vector (si-circ-0013561, siRNA concentration 20 nM, siRNA for circ-0013561: 5′-TTTAGGTCTTGGGCTTGCC-3′), and ANXA2 overexpression vector (ANXA2, final concentration: 20 nM, cDNA sequence cloned into pcDNA3.1 vector) purchased from RiboBio (Guangzhou, China) were used for transfection with Lipofectamine 2000 (Thermo Fisher Scientific, Waltham, MA, USA).

### Western blot

Proteins from lysed cells (50 μg) were separated by 10% SDS-PAGE and transferred to nitrocellulose membranes, and this was followed by blocking for 2 h. Next, membranes were incubated overnight with primary antibodies followed by horseradish peroxidase-conjugated secondary antibodies. The protein bands were visualized with ECL Plus Detection Reagent (Applygen, Beijing, China).

### Cell migration assay

We used 24-well Transwell chambers (8 µm pore membrane, BD Biosciences, Franklin Lakes, NJ, USA) for cell migration analysis. We plated SKOV3 and A2780 cells (200 µL serum-free DMEM medium including 1 × 10^5^ cells) into the upper chamber and 500 µL DMEM medium with 20% FBS into the bottom chamber. Then cells were cultured for 1 day at 37 °C. After fixing with 4% polyformaldehyde and staining with 0.1% crystal violet (Shanghai Yisheng Biotechnology, Shanghai, China) at room temperature for 10 min, the cell numbers of the bottom chamber were calculated. We photographed the cells using a Zeiss Axio Observer D1 microscope (magnification, ×200).

### 5-ethynyl-20-deoxyuridine (EdU) analysis

We used EdU assay kits (Thermo Fisher Scientific) for cell proliferation analysis. SKOV3 and A2780 cells (1 × 10^5^) were maintained in 6-well plates for 2 days before adding 100 μL EdU to the wells for 2 h. Then cells were fixed with 4% formaldehyde followed by 0.3% Triton X-100 for 10 min. We analyzed EdU-positive cells using a Zeiss LSM 700 confocal microscope.

### Cell counting kit-8 (CCK8) assay

We incubated SKOV3 and A2780 cells in 10% CCK8 diluent in a normal culture medium at 37 °C. We determined proliferation rates at 0-, 1-, 2-, and 3-day post-transfection. We detected the absorbance of each well using a microplate reader.

### RT-qPCR

Total RNA from cells or tissues was isolated using the TRIzol reagent kit (Invitrogen, Carlsbad, CA, USA). cDNA was synthesized and amplified using the TaqMan miRNA reverse transcription kit (Thermo Fisher Scientific), and we performed qPCR by applying the TaqMan assay kit (Applied Biosystems, Foster City, CA, USA). We used the 2^−ΔΔCT^ method to measure relative expression fold change, with *U6* and *GAPDH* as internal references. Primers utilized in the study were as follows:

hsa_circ_0013561: forward 5′-GAATGCTGTCCTGTCCTC-3′, reverse 5′-TGCCAATCATGGTCAGAG-3′;

miR-23b-3p: forward 5′-CGGGCATCACATTGCCAGG-3′, reverse: 5′-CAGCCACAAAAGAGCACAAT-3′;

*ANXA2*: forward: 5′-TTGCTGATCGGCTGTATG-3′, reverse: 5′-GGTAGTCGCCCTTAGTGTC-3′;

*U6*: forward: 5′-CTCGCTTCGGCAGCACA-3′ and reverse: 5′-AACGCTTCACGAATTTGCGT-3′;

*GAPDH*: forward, 5′-AATGGGCAGCCGTTAGGAAA-3′; reverse: 5′-TGAAGGGGTCATTGATGGCA-3′.

### RNA immunoprecipitation (RIP) assay

For the RIP assay, a Magna RIP kit (Millipore, Billerica, MA, USA) was used. Briefly, cell lysate was treated with RIP buffer containing magnetic beads conjugated with human anti-Ago2 antibody (Millipore, Billerica, MA, USA), or negative control IgG. Beads were washed with wash buffer, and the complexes were incubated with 0.1% SDS/proteinase K to remove proteins. qRT-PCR assay was carried out afterward.

### Dual-luciferase reporter assay

The wild type (Wt) or mutant type (Mut) hsa_circ_0013561 or ANXA2 3’-untranslated region (3’ UTR) sequences containing putative miR-23b-3p binding sites were cloned into the psi-CHECK vector (Promega, Madison, Wisconsin, USA). We named these clones as circ-0013561-Wt, ANXA2-Wt, circ-0013561-Mut, and ANXA2-Mut, respectively. The firefly luciferase signal regulated by the 3’ UTR of the target gene was normalized to the renilla luciferase signal. The HEK293T cell transfection was performed using Lipofectamine 2000. We measured renilla and firefly luciferase activities 2 days after transfection by applying a luminometer (Molecular Devices, San Jose, CA, USA).

### Tumor xenograft models

To construct a subcutaneous OC model, viable Wt or sh-circ-0013561 SKOV3 cells (2 × 10^6^) were injected into the right flanks of nude mice. We measured tumor volumes every 5 days for 30 consecutive days using a Vernier caliper. The tumor volume was calculated as length × width^2^ × 0.5. We also measured the relative expression of cancer marker Ki67 using immunohistochemical methods.

For the metastasis analysis in vivo, luminescence-labeled SKOV3 cells were transfected stably with the negative control (NC). We suspended sh-circ-0013561 in sterile phosphate-buffered saline, which was injected into the tail vein of each nude mouse. After 4 weeks, we evaluated lung metastasis using an in vivo bioluminescence imaging system. We counted the number of metastatic foci in lung tissues following hematoxylin and eosin (HE) staining.

### Statistical analysis

Statistical analysis was conducted using GraphPad Prism 7.0 (GraphPad, La Jolla, CA, USA). Data were analyzed by student’s *t*-test, Chi-square test, and Pearson’s correlation test as appropriate. Quantitative data were presented as mean ± standard deviation (SD). *P*-value < 0.05 was considered as statistical significance.

## Results

### Hsa_circ_0013561 is upregulated in OC and associated with poor prognosis

**Fig. 1:**
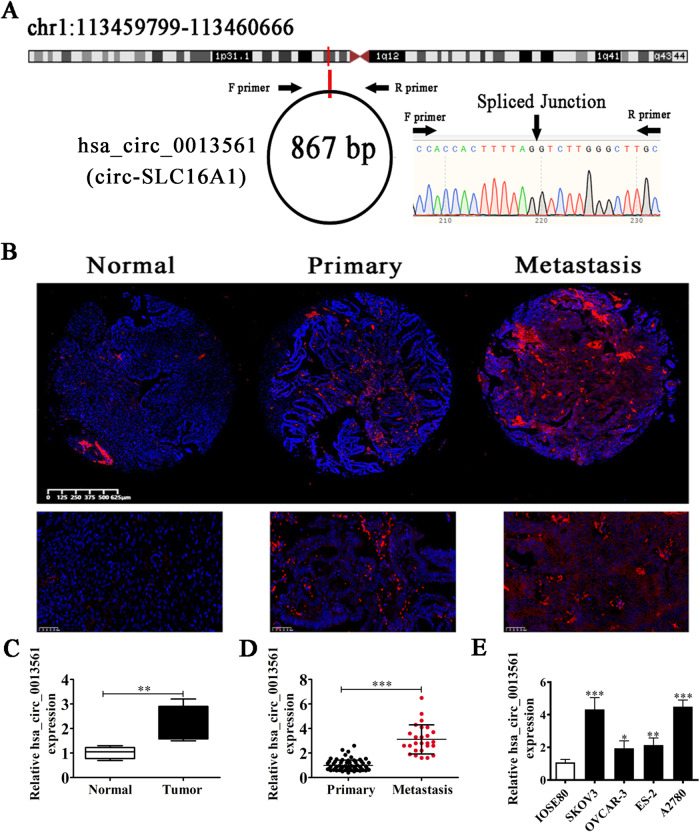
The characterization and expression of hsa_circ_0013561 in OC. **A** Schematic diagram illustrating the formation of hsa_circ_0013561. Hsa_circ_0013561 was back-spliced from an exon of *SLC16A1*, and its back-splicing junction was confirmed by Sanger sequencing. **B** FISH detection showed the expression and subcellular localization of hsa_circ_0013561 in normal, primary, and metastatic OC tissues. **C** The expression level of hsa_circ_0013561 in tumor tissues and normal controls, measured by FISH, **P < 0.01 vs. Normal. **D** The expression level of hsa_circ_0013561 in primary and metastatic OC tissues, measured by FISH, ***P < 0.001 vs. metastasis. **E** RT-qPCR assay showed the expression of hsa_circ_0013561 in normal ovarian cell (IOSE80) and different OC cells (SKOV3, OVCAR-3, ES-2, and A2780). *P < 0.05, **P < 0.01, ***P < 0.001 OC cells vs. IOSE80.

Our preliminary experiment discovered that hsa_circ_0013561 was aberrantly expressed in OC. Bioinformatics data [12] showed that hsa_circ_0013561 (Circbank [13] ID: hsa_circSLC16A1_004) is derived from the Solute Carrier Family 16 Member 1 (*SLC16A1*) gene. This host gene located on chr1:113459799-113460666 retrieved from UCSC Genome Browser (GRCh37/hg19) [14]. Hsa_circ_0013561 is formed by cyclization of exon 4 (867 base pairs) from *SLC16A1* [15] (Fig. 1A). Sanger sequencing confirmed the sequences spanning the back-splicing junction (BSJ) of hsa_circ_0013561 which were also concordant with that in circBase. FISH data indicated that hsa_circ_0013561 was mainly localized in the cytoplasm (Fig. 1B).

Hsa_circ_0013561 expression was higher in primary OC tissues compared to normal tissues (Fig. 1C), and its expression increased in metastatic OC tissues compared with both normal and primary OC tissues (Fig. 1D). The RT-qPCR results showed that hsa_circ_0013561 expression was higher in OC cell lines SKOV3, OVCAR-3, ES-2, and A2780 compared with IOSE80. Also, SKOV3 and A2780 cells had higher hsa_circ_0013561 expression compared to OCVAR-3 and ES-2 cells (Fig. 1E). Therefore, A2780 and SKOV3 cells were used in the subsequent experiments.

### Hsa_circ_0013561 downregulation inhibits OC cell proliferation and tumor growth

**Fig. 2:**
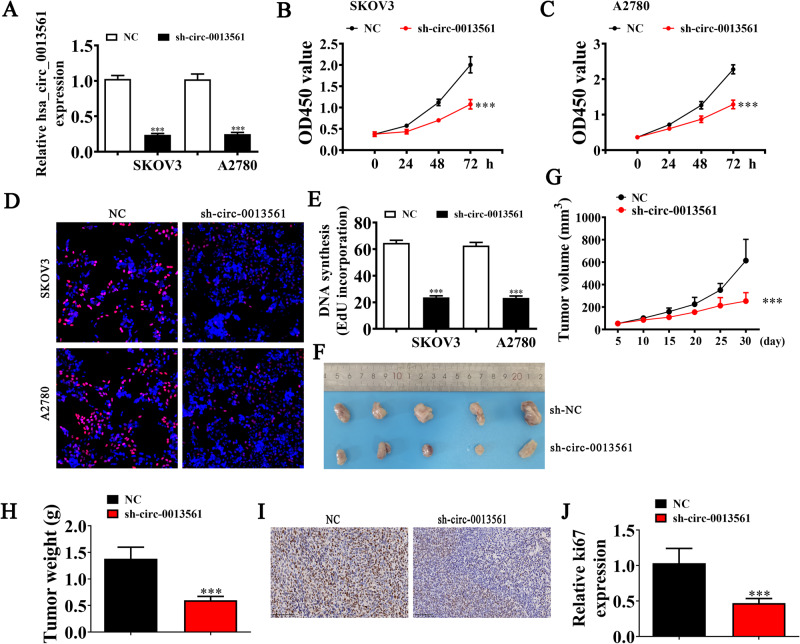
Hsa_circ_0013561 downregulation inhibits OC cell proliferation and tumor growth. **A** The expression of hsa_circ_0013561 in SKOV3 and A2780 transfected with hsa_circ_0013561 silencing vector (sh-circ-0013561) or control vector (NC), measured by RT-qPCR, ***P < 0.001 vs. NC. **B**, **C** The proliferation capacity of SKOV3 and A2780 was detected by CCK8, ***P < 0.001 vs. NC. **D**, **E** The proliferation capacity of SKOV3 and A2780, detected by EdU, ***P < 0.001 vs. NC. **F** Representative photographs of SKOV3 tumor formation in xenograft model of nude mice. **G**, **H** Summary of tumor volumes and weights in subcutaneous tumor xenograft model, ***P < 0.001 vs. sh-NC. **I**, **J** The relative number of Ki-67 positive cells in subcutaneous xenograft model, measured by immunohistochemical scores, ^***^P < 0.001 vs. NC.

In order to explore the biological functions of hsa_circ_0013561, we performed phenotypic experiments in vitro and in vivo. First, we constructed the siRNA specifically targeting the BSJ of hsa_circ_0013561. After transfecting SKOV3 and A2780 cells with siRNA against hsa_circ_0013561, RT-qPCR showed that the expression level of hsa_circ_0013561 significantly decreased relative to that of negative control (Fig. 2A).

Then, through CCK8 (Fig. 2B, C) and EdU (Fig. 2D, E) assays, we found that hsa_circ_0013561 silencing significantly inhibited the proliferation in SKOV3 and A2780 cells. Furthermore, the in vivo xenograft mice assay showed that tumor weight and volume (Fig. 2F–H) in nude mice injected with SKOV3 cells markedly decreased post-si-circ-0013561 transfection. Immunohistochemical analysis also showed that the downregulation of hsa_circ_0013561 significantly inhibited Ki67 expression in tumor tissues (Fig. 2I, J). Collectively, these results suggested downregulation of hsa_circ_0013561 suppresses OC cell proliferation and tumor growth.

### Hsa_circ_0013561 downregulation inhibits OC cell migration and tumor metastasis

**Fig. 3:**
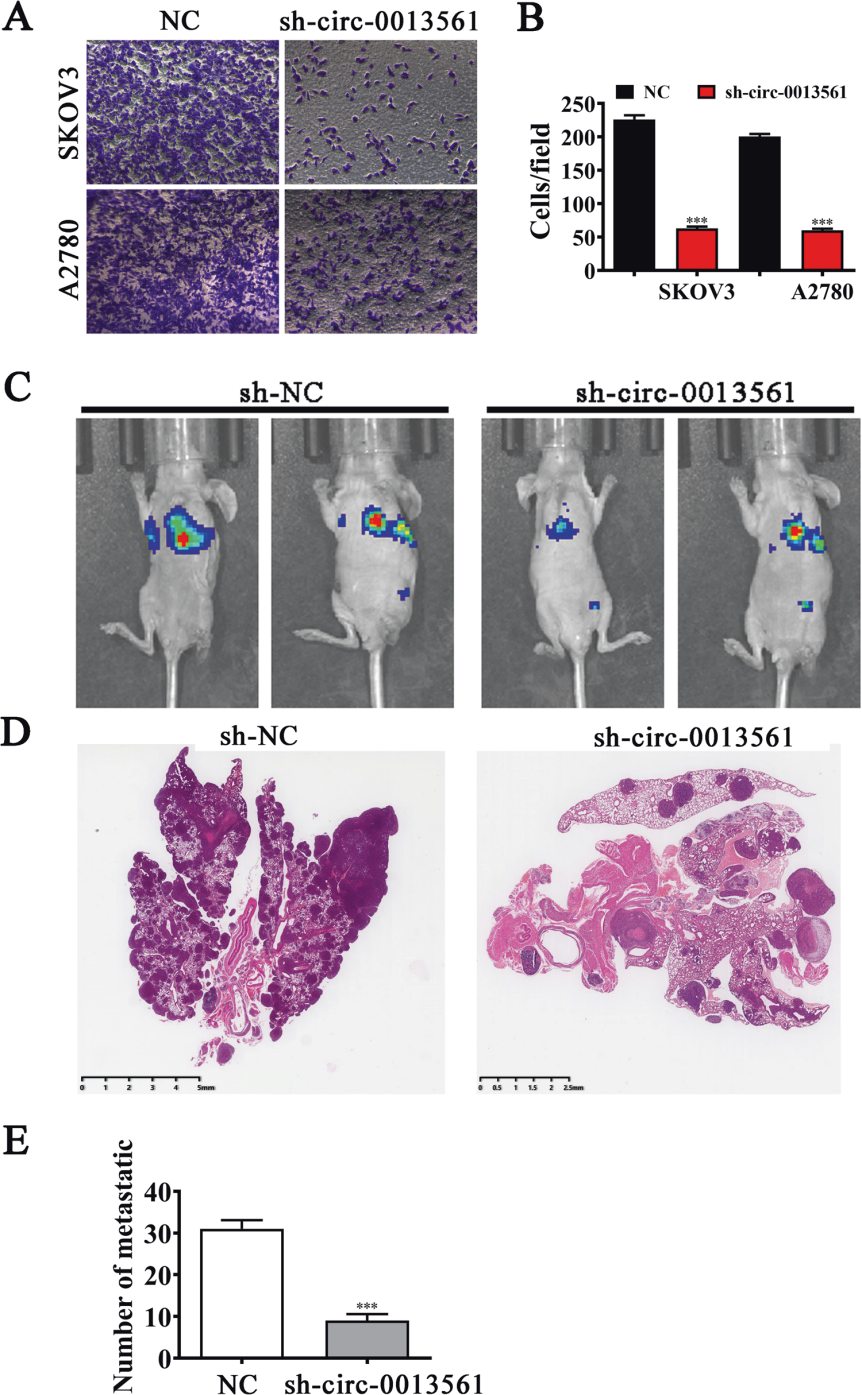
Hsa_circ_0013561 downregulation inhibits OC cell migration and tumor metastasis. **A**, **B** The migration capacity of SKOV3 and A2780 was detected by transwell, ***P < 0.001 vs. NC. **C** The pulmonary metastasis capacity of SKOV3 cells was investigated by live imaging. **D**, **E** The number of metastatic foci in lung tissues was calculated under a microscope based on HE staining, ^***^P < 0.001 vs. NC.

**Fig. 4:**
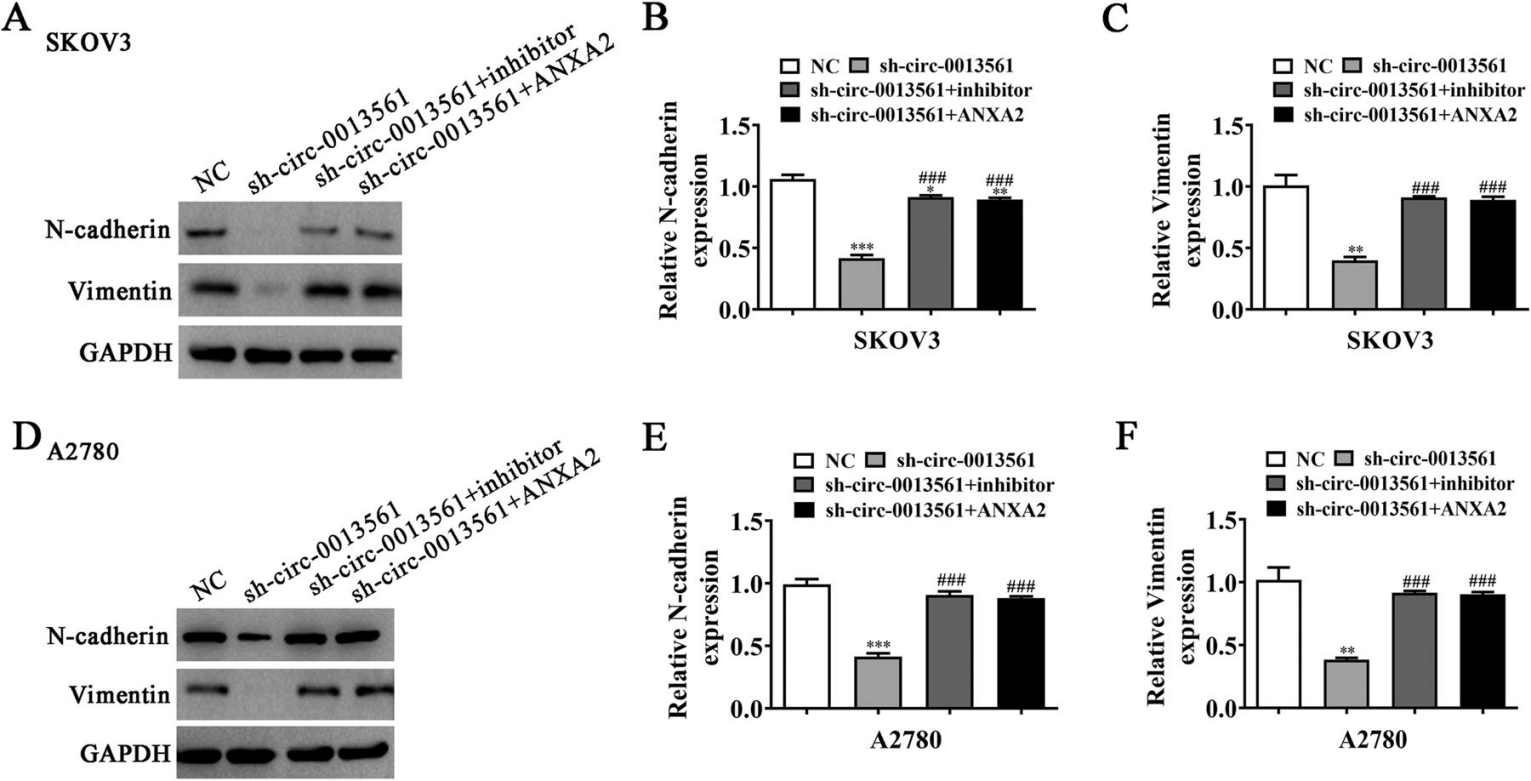
MiR-23b-3p inhibition or ANXA2 overexpression reverses the expression of EMT-affiliated proteins in OC cells post-hsa_circ_0013561 silencing. **A**–**C** The expression of N-cadherin and Vimentin in SKOV3, measured by Western blot, *P < 0.05, **P < 0.01, ***P < 0.001 vs. NC; ^###^*P* < 0.001 vs. sh-circ-0013561. **D**–**F** The expression of N-cadherin and Vimentin in A2780, measured by Western blot, **P < 0.01, ***P < 0.001 vs. NC; ^###^*P* < 0.001 vs. sh-circ-0013561.

Transwell assays showed that hsa_circ_0013561 downregulation significantly inhibited the invasion and migration in SKOV3 and A2780 cells (Fig. 3A, B). In order to explore the impacts of hsa_circ_0013561 on OC cell migration in vivo, we established a mouse lung-metastasis model. Live imaging showed that hsa_circ_0013561 silencing also significantly decreased the pulmonary metastasis capacity of SKOV3 cells (Fig. 3C). In addition, HE staining was applied to demonstrate the number and histopathological characteristics of metastatic foci in lung tissues. The HE results showed that hsa_circ_0013561 silencing significantly reduced the number of lung metastatic foci (Fig. 3D). Thus, the above results suggested that the downregulated hsa_circ_0013561 inhibits OC cell migration and tumor metastasis. Besides, it is noteworthy that hsa_circ_0013561 silencing markedly suppressed N-cadherin and Vimentin in SKOV3 and A2780 cells (Fig. 4A–F). This finding indicated that hsa_circ_0013561 may act as an inducer of EMT in OC.

### MiR-23b-3p and ANXA2 are downstream hsa_circ_0013561 targets

**Fig. 5:**
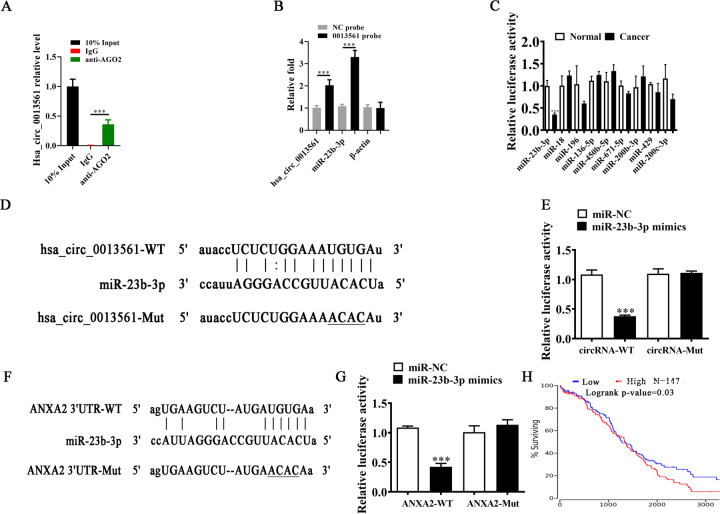
MiR-23b-3p and ANXA2 are the downstream targets of hsa_circ_0013561. **A** AGO2 RNA-binding protein immunoprecipitation. All data are shown as the mean ± SD. ***P < 0.001 compared to IgG. **B** circRNA pull-down assay. 0013561 is referred to as hsa_circ_0013561. **P < 0.01 compared to negative control (NC) probe. **C** The luciferase activity of HEK293T cells with hsa_circ_0013561-Wt or hsa_circ_0013561-Mut co-transfected with different miRNA mimics or control (NC) mimics. **D** Prediction of miR-23b-3p binding sites in hsa_circ_0013561. The mutated (Mut) version of hsa_circ_0013561 was constructed. **E** Relative luciferase activity was tested in HEK293T cells with hsa_circ_0013561-Wt or hsa_circ_0013561-Mut co-transfected with miR-23b-3p mimics or NC mimics, ***P < 0.001. **F** Prediction of miR-23b-3p binding sites within the 3’ UTR of ANXA2. The Mut version of ANXA2 3’-UTR is shown. **G** Relative luciferase activity was tested in HEK293T cells with ANXA2 3’ UTR-Wt or ANXA2 3’ UTR-Mut co-transfected with miR-23b-3p mimics or NC mimics, ***P < 0.001. **H** Bioinformatics analysis (http://www.oncolnc.org/) showed the survival rate between OC patients with high and low ANXA2 expression.

To validate this result, we conducted AGO2 RIP and found that endogenous hsa_circ_0013561 could be specifically pulled down by anti-AGO2 antibody (Fig. 5A). This suggests that hsa_circ_0013561 acts as a miRNA-binding partner. By using a probe targeting the hsa_circ_0013561 junction site, we found that hsa_circ_0013561 and miR-23b-3p were significantly more abundant compared with the control (Fig. 5B).

To investigate the regulatory role of hsa_circ_0013561, bioinformatics analysis in conjunction with luciferase reporter assay was performed. First, we used circRNA-miRNA prediction databases, the circBank (http://www.circbank.cn/index.html) [13] and circMine (http://hpcc.siat.ac.cn/circmine/home) [16], to explore the potential miRNAs that interact with hsa_circ_0013561. A series of miRNAs, such as miR-23b-3p, miR-136-5p, and miR-671-5p, were screened out as the candidate targets of hsa_circ_0013561 in both databases. Then, we conducted a dual-luciferase reporter assay in which HEK293T cells with hsa_circ_0013561-Wt or hsa_circ_0013561-Mut were co-transfected with different miRNA mimics or control (NC) mimics. The results demonstrated that miR-23b-3p significantly inhibited the fluorescein intensity, indicating that miR-23b-3p interacts with hsa_circ_0013561 (Fig. 5A). Next, luciferase reporter analyses confirmed that miR-23b-3p could markedly reduce the luciferase activity in Wt cells, but not in Mut cells (Fig. 5B, C), thereby indicating that miR-23b-3p is a downstream target of hsa_circ_0013561.

We further explored the potential genes regulated by miR-23b-3p. The starBase (https://starbase.sysu.edu.cn/starbase2/) [17], miRbase (https://www.mirbase.org/) [18], miRDB (https://mirdb.org/) [19], and TargetScan (https://www.targetscan.org/vert_72/) [20] were applied to predict candidate genes. Then we filtered candidates by taking intersection and identified ANXA2 as a potential target regulated by miR-23b-3p. To confirm the interactive relationship between miR-23b-3p and ANXA2, Mut/Wt ANXA2 3’ UTR sequences containing miR-23b-3p binding sites were constructed into luciferase reporter vector (Fig. 5D). Luciferase reporter vectors were transfected into HEK293T cells with or without the miR-23b-3p mimics. Also, the results showed that miR-23b-3p significantly inhibited the luciferase activity in Wt cells, compared with that in Mut cells (Fig. 5E), indicating that ANXA2 was the miR-23b-3p target. Bioinformatics analysis (http://www.oncolnc.org/) conducted for overall survival analysis showed that the elevated ANXA2 expression in OC was associated with a worse prognosis (Fig. 5F).

### MiR-23b-3p inhibition or ANXA2 overexpression reverses OC cell proliferation, migration, and EMT post-hsa_circ_0013561 silencing

**Fig. 6:**
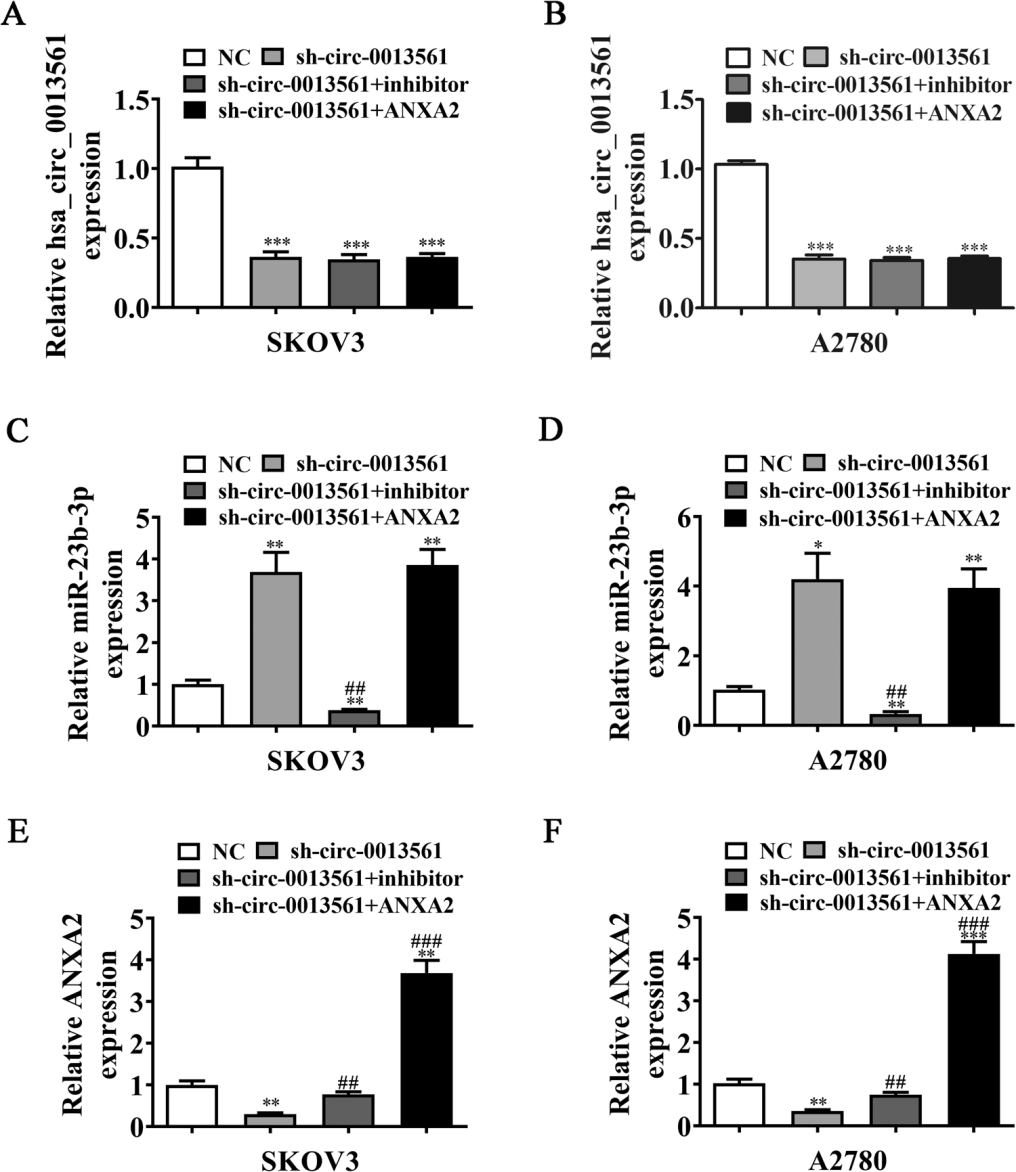
Intermolecular regulation among hsa_circ_0013561, miR-23b-3p, and ANXA2. **A**–**F** A series of rescue experiments demonstrated the expression of hsa_circ_0013561, miR-23b-3p, and ANXA2 in SKOV3 and A2780 after transfection with si-circ-0013561, miR-23b-3p inhibitor, and ANXA2 overexpression vector singly or combined, measured by RT-qPCR, *P < 0.05, **P < 0.01, ***P < 0.001 vs. NC; ^##^*P* < 0.01, ^###^*P* < 0.001 vs. si-circ-0013561.

RT-qPCR data demonstrated that the hsa_circ_0013561 expression decreased after transfection with the hsa_circ_0013561 silencing vector, whereas miR-23b-3p inhibition or ANXA2 overexpression had no effects on the hsa_circ_0013561 expression in SKOV3 and A2780 cells (Fig. 6A, B). This result suggested that both miR-23b-3p and ANXA2 are the downstream molecules of hsa_circ_0013561. Further, hsa_circ_0013561 silencing increased the miR-23b-3p expression, but ANXA2 overexpression had no effect on si-circ-0013561-induced miR-23b-3p expression (Fig. 6C, D). This finding suggested that miR-23b-3p is regulated by hsa_circ_0013561 and is the molecule upstream of ANXA2. Moreover, hsa_circ_0013561 silencing decreased the ANXA2 expression, but miR-23b-3p inhibition reversed the suppressive effects of si-circ-0013561 on ANXA2 expression. After transfection of the ANXA2 overexpression vector, the ANXA2 expression increased significantly (Fig. 6E, F), indicating that hsa_circ_0013561 enhances the ANXA2 expression via miR-23b-3p.

**Fig. 7:**
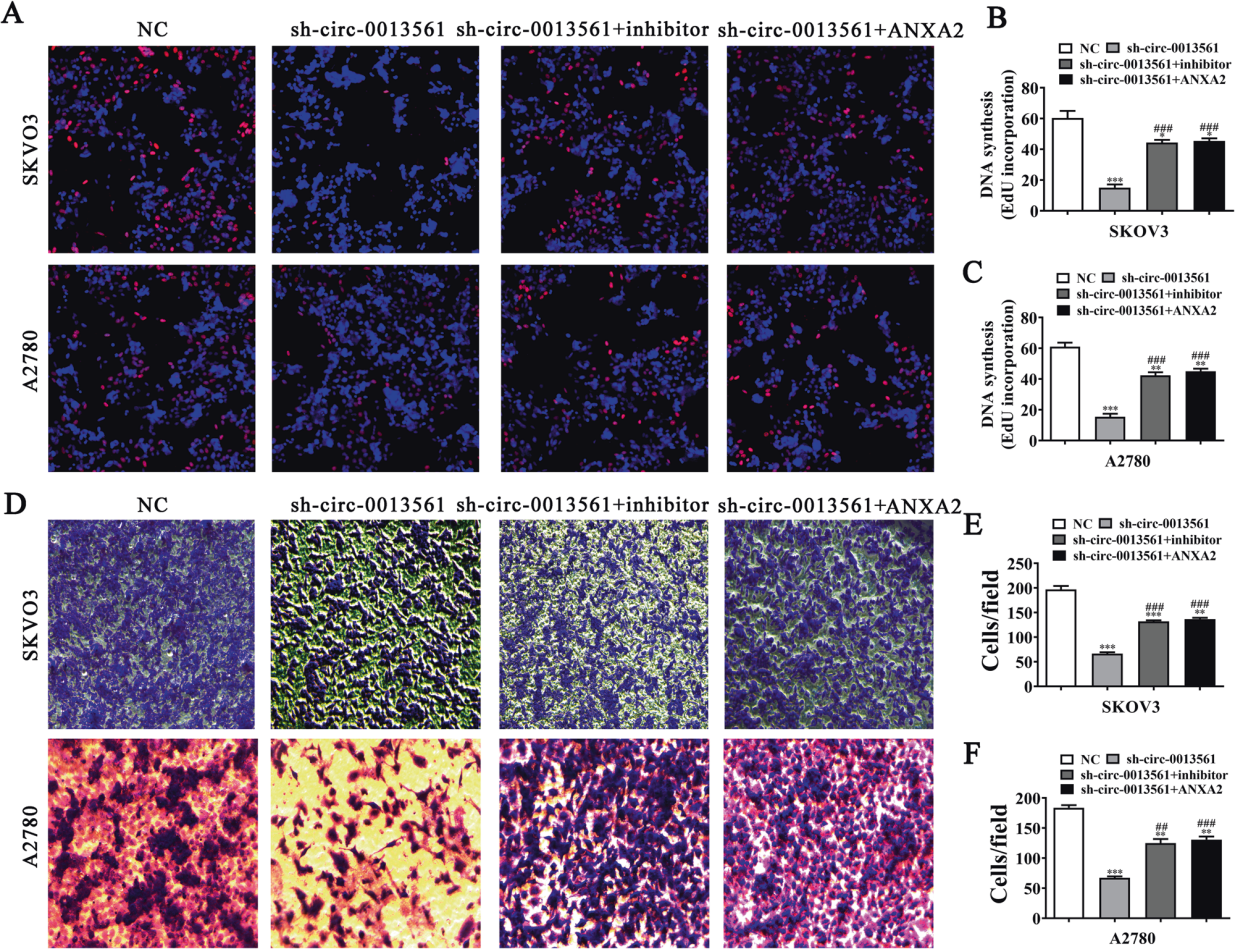
MiR-23b-3p inhibition or ANXA2 overexpression reverses OC cell proliferation and migration post-hsa_circ_0013561 silencing. **A**–**C** The proliferation capacity of SKOV3 and A2780 detected by EdU assay, *P < 0.05, **P < 0.01, ***P < 0.001 vs. NC; ^###^*P* < 0.001 vs. sh-circ-0013561. **D**–**F** The invasion and migration capacity of SKOV3 and A2780 detected by transwell assay, **P < 0.01, ***P < 0.001 vs. NC; ^##^*P* < 0.01, ^###^*P* < 0.001 vs. sh-circ-0013561.

The EdU assay showed that miR-23b-3p inhibition or ANXA2 overexpression restored the proliferation capacity in SKOV3 and A2780 cells post-hsa_circ_0013561 silencing (Fig. 6A–C). In addition, the transwell migration assay showed that miR-23b-3p inhibition or ANXA2 overexpression resumed the OC cell migration post-hsa_circ_0013561 silencing (Fig. 7D–F). Western blot detection revealed that miR-23b-3p inhibition or ANXA2 overexpression resumed the expression of EMT-associated proteins, N-cadherin and Vimentin, in SKOV3 and A2780 cells post-hsa_circ_0013561 silencing (Fig. 4).

### ANXA2 overexpression reverses OC cell proliferation, migration, and EMT after miR-23b-3p upregulation

**Fig. 8:**
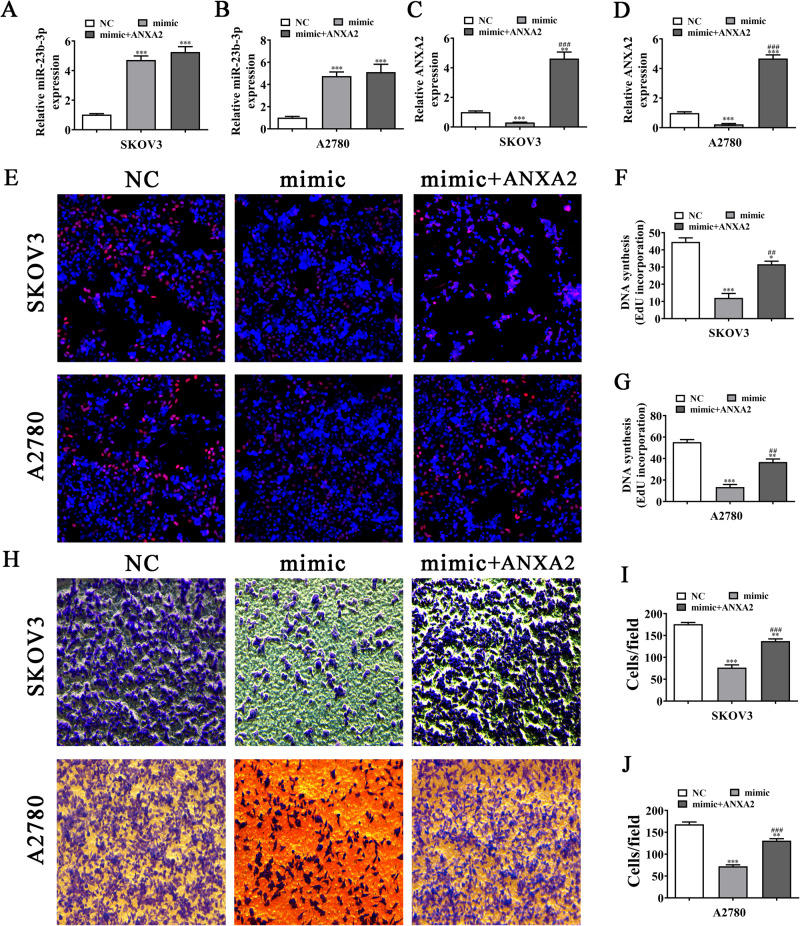
ANXA2 overexpression reverses OC cell proliferation and migration after miR-23b-3p upregulation. **A**–**D** The expression of miR-23b-3p and ANXA2 in SKOV3 and A2780 detected by RT-qPCR, **P < 0.01, ***P < 0.001 vs. NC; ^###^*P* < 0.001 vs. miR-23b-3p mimic. **E**–**G** The proliferation capacity of SKOV3 and A2780 detected by EdU assay, *P < 0.05, **P < 0.01, ***P < 0.001 vs. NC; ^##^*P* < 0.01 vs. miR-23b-3p mimic. **H**–**J** The invasion and migration capacity of SKOV3 and A2780 detected by transwell assay, **P < 0.01, ***P < 0.001 vs. NC; ^###^*P* < 0.001 vs. miR-23b-3p mimic.

We found that miR-23b-3p overexpression promoted the miR-23b-3p expression in SKOV3 and A2780 cells and that ANXA2 upregulation could not reverse the miR-23b-3p expression (Fig. 8A, B). In addition, miR-23b-3p overexpression inhibited the ANXA2 expression. However, after transfection with the heterologous vector of ANXA2 overexpression, the ANXA2 expression significantly increased in SKOV3 and A2780 cells (Fig. 8C, D).

**Fig. 9:**
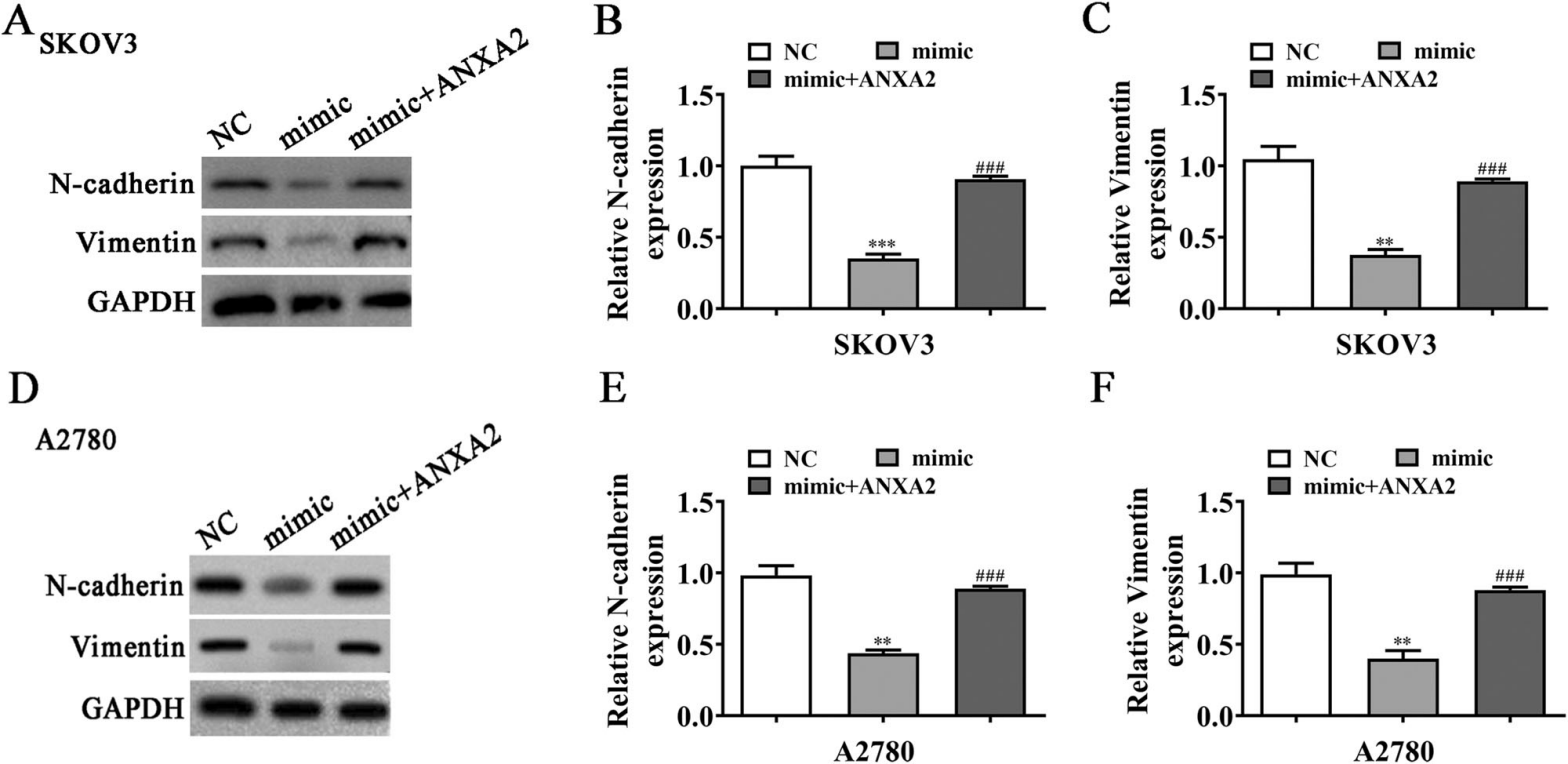
ANXA2 overexpression reverses the expression of EMT-affiliated proteins in OC cells after miR-23b-3p upregulation. **A**–**C** The expression of N-cadherin and Vimentin in SKOV3, measured by Western blot, **P < 0.01, ***P < 0.001 vs. NC; ^###^*P* < 0.001 vs. mimic. **D**–**F** The expression of N-cadherin and Vimentin in A2780, measured by Western blot, **P < 0.01 vs. NC; ^###^*P* < 0.001 vs. mimic.

The EdU assay showed that ANXA2 overexpression resumed the proliferation ability of SKOV3 and A2780 cells post-miR-23b-3p upregulation (Fig. 8E–G). Transwell migration data also showed that ANXA2 overexpression restored the OC cell migration post-miR-23b-3p upregulation (Fig. 8H–J). Additionally, Western blot detection demonstrated that ANXA2 overexpression restored the expression of EMT-associated proteins, N-cadherin and Vimentin, in SKOV3 and A2780 cells post-miR-23b-3p upregulation (Fig. 9).

## Discussion

Accumulating evidence confirms that circRNAs play an important part in the progression of OC [6, 10, 11]. In the current study, we discovered that the hsa_circ_0013561 expression significantly increased in OC tissues, especially during metastasis, suggesting that high hsa_circ_0013561 expression is associated with poor prognosis. We also observed the same trend in OC cell lines and their normal controls, among which SKOV3 and A2780 cells had the highest hsa_circ_0013561 levels. Thus, subsequent functional assays were conducted using the hsa_circ_0013561 silencing vector in these two cell lines. The observations showed that downregulation of hsa_circ_0013561 substantially inhibited the OC cell proliferation, migration, tumor growth, tumor metastasis, as well as mesenchymal markers. The results above suggested that hsa_circ_0013561 could serve as a new potential oncogenic circular RNA in the proliferation and aggression of OC. In addition, it may also function as an EMT regulator to exhibit pro-tumor activities.

To mechanistically understand the regulatory role of hsa_circ_0013561 in OC progression, bioinformatics analyses and in vitro and in vivo experiments were performed. We found that MiR-23b-3p was the downstream target of hsa_circ_0013561. Hsa_circ_0013561 silencing increased the miR-23b-3p expression, and miR-23b-3p inhibition restored OC cell proliferation and migration capacities post-hsa_circ_0013561 silencing. These results showed that miR-23b-3p represses OC progression and is regulated by hsa_circ_0013561. So far, the potential roles of miR-23b-3p in different cancers have been much reported in the literature, indicating miR-23b-3p as a tumor suppressor [21, 22]. However, the impacts of miR-23b-3p on OC have been rarely investigated. The only two experimental studies in this field are focused on long noncoding RNAs (lncRNAs). Yang et al. found that LINC00909 enhances OC progression via the miR-23b-3p/MRC2 Axis [23], while Lin H et al. described a positive feedback loop of LncRNA CASC15/miR-23b-3p & miR-24-3p/SMAD3 to promote EMT and metastasis in OC [24]. Our investigation revealed that miR-23b-3p exerts an anti-tumor effect, which is consistent with previous studies.

As a calcium-dependent membrane-binding and phospholipid-binding protein, ANXA2 plays multiple roles in various biological processes. For example, it functions to stimulate fibrinolytic activity, degrade extracellular matrix, promote angiogenesis, and connect membrane-cytoskeleton or membrane-membrane [25–27]. Recent data showed that ANXA2 is expressed aberrantly in various malignancies [26, 27]. In addition, it generally correlates with EMT, portending a poor prognosis in many cancers [28–30]. However, the molecular mechanisms of ANXA2 in the process of EMT remain unknown. In this study, we found that ANXA2 was the downstream target of both hsa_circ_0013561 and miR-23b-3p. Previous studies have confirmed that miR-23b-3p could bind specifically to the 3’ untranslated region of ANXA2 in pancreatic ductal adenocarcinoma [31]. This study found that ANXA2 overexpression rescued OC cell proliferation, migration, and EMT post-hsa_circ_0013561 silencing. Moreover, ANXA2 overexpression also reversed OC cell migration, proliferation, and EMT after miR-23b-3p upregulation. These results verified that ANXA2 could act as an EMT-facilitated protein in OC, and described for the first time that hsa_circ_0013561 increases the expression of ANXA2 through regulating miR-23b-3p competitively, resulting in EMT and metastasis of OC.

## Conclusion

In summary, our data demonstrate that hsa_circ_0013561 promotes EMT and tumor progression via the miR-23b-3p/ANXA2 axis. Thus, hsa_circ_0013561 may serve as a novel oncogenic biomarker and potential therapeutic target in OC progression.

## Data Availability

The datasets used and/or analyzed during the current study are available from the corresponding author upon reasonable request.
